# Immunomodulatory drugs in multiple myeloma: Impact of the SCARMET (Self CARe and MEdication Toxicity) educational intervention on outpatients’ knowledge to manage adverse effects

**DOI:** 10.1371/journal.pone.0243309

**Published:** 2020-12-04

**Authors:** Juliette Périchou, Florence Ranchon, Chloé Herledan, Laure Huot, Virginie Larbre, Isabelle Carpentier, Anne Lazareth, Lionel Karlin, Karen Beny, Nicolas Vantard, Vérane Schwiertz, Anne Gaelle Caffin, Amandine Baudouin, Pierre Sesques, Gabriel Brisou, Hervé Ghesquières, Gilles Salles, Catherine Rioufol

**Affiliations:** 1 Unité de Pharmacie Clinique Oncologique, Groupement Hospitalier Sud, Hospices Civils de Lyon, Pierre-Bénite, France; 2 Université Lyon 1- EMR 3738, Lyon, France; 3 Département de la Recherche Clinique et de l’Innovation, Cellule Innovation, Hospices Civils de Lyon, Lyon, France; 4 Pharmacie Centrale, Hospices Civils de Lyon, Pierre-Bénite, France; 5 Hematology Department, Groupement Hospitalier Sud, Hospices Civils de Lyon, Pierre-Bénite, France; 6 Centre de Recherche en Cancérologie de Lyon, INSERM 1052 CNRS 5286, Université Lyon 1, Lyon, France; University of South Australia, AUSTRALIA

## Abstract

Long-term multiple myeloma therapy by immunomodulatory drugs (IMiDs) raises the question of management of adverse effects. The aim of this study is to assess the impact of an educational session for patients on the acquisition of knowledge to manage hematologic and thromboembolic adverse effects of IMiDs. In this prospective single-center study, patients attended an educational session with a hospital clinical pharmacist and a nurse. The primary endpoint was the patient’s level of knowledge for the management of IMiDs adverse effects, assess with a dedicated questionnaire administered before the session then 1 and 6 months after. Assessment of knowledge was combined with self-assessment of certainty. The secondary endpoints were adherence and IMiD treatment satisfaction. 50 patients were included. Patient knowledge increased at 1 month (p<0.001) despite a loss of knowledge at 6 months (p<0.05). Six months after the educational intervention, the number of patients with skills considered satisfactory by the pharmacist and nurse increased (p<0.01). Most patients showed satisfactory adherence, with medication possession ratio ≥ 80%. The Self CARe and MEdication Toxicity (SCARMET) study highlighted the impact of multidisciplinary follow-up in multiple myeloma patients to improve knowledge of toxicity self-management.

## Introduction

Multiple myeloma is one of the most common hematologic malignancies, with 32,270 estimated incident cases in the United States in 2020, corresponding to 1.8% of all new cancer cases [1]. Clinical outcome is heterogeneous and survival ranges from a few months to more than 10 years, depending on host factors, tumor burden, cytogenetic abnormalities and therapeutic response [2]. Immunomodulatory drugs (IMiDs) have become a cornerstone of treatment, increasing response rates to >70% [3].

Oral anticancer treatment is well accepted, thanks to easy administration and improved autonomy and comfort, with less time spent in hospital than with intravenous treatment [4]. Nevertheless, many severe Adverse Drug Reactions (ADR) are reported with IMiDs, including gastrointestinal disorders, peripheral neuropathy, Venous Thrombotic Events (VTE), and frequent hematologic toxicity [5–7]. Other drug-related problems associated with oral anticancer therapies are worrisome, with complex medication regimens, inappropriate management of ADR or drug interactions [8] and a lack of communication between health-care providers, with risk of medication error [9]. All these problems most commonly result in non-adherence to the recommended treatment plan [10], impairing the expected dose-efficacy response relationship. Several recent studies have focused on adherence to oral agents in patients with cancer, but this topic remains under-documented in multiple myeloma patients [11, 12].

As long-term oral treatment for multiple myeloma is growing, strategies to manage and prevent treatment-related toxicities are a major concern. Patient education approaches have been developed, to increase self-care knowledge and skills, enabling autonomy in self-administration of medication [13]. Acquisition of self-care skills is estimated by measuring the capacity of the patient to solve problems [14, 15]. Patients’ certainty in their knowledge should be further explored, because trust or doubt about illness and treatment may influence the decision to take action [16]. This approach distinguishes usable knowledge (both correct and sure), unusable knowledge (correct but insufficiently sure as a basis for action) and harmful conviction (incorrect but “sure”) [17, 18].

A real life multidisciplinary care plan for cancer outpatients, named ONCORAL (ONCological care for outpatients with ORAL anticancer drugs), was set up, based on educational interviews. A specific study on multiple myeloma patients, the Self-CARe and MEdication Toxicity (SCARMET) program, was drawn to optimize self-management of IMiDs ADR. The aims of the present study are first to assess the impact of the SCARMET program on the acquisition of knowledge on ADR self-management skills, and secondary on adherence and satisfaction to IMiDs treatment.

## Method

### Population

All patients with multiple myeloma treated with IMiDs (thalidomide, lenalidomide, pomalidomide) from October 2015 to October 2016 in the Hematology department of Lyon Sud University Hospital (Hospices Civils de Lyon, Lyon, France) were invited to receive the multidisciplinary care plan ONCORAL and to participate in the SCARMET study. Patients who were not French-speaking or had difficulties of communication, were living in institutions or receiving home help by a professional caregiver for treatment were not included. To allow the assessment knowledge at 6 months, patients should always be treated with IMiDs at this time point. Patients were informed of the purpose of the study, a detailed information leaflet was given to them, and their consent was collected. The study respected the Helsinki Declaration, French regulations, and institutional guidelines.

### SCARMET program

The SCARMET program was drawn up by a panel of experts made up of two hematologists, three clinical pharmacists and one nurse, all of them specialized in cancer care. The educational intervention was conducted at the end of the hematologist consultation, by an educational team composed of a nurse and a hospital clinical pharmacist, working together with the hematologist. The pharmacist discussed the individual treatment plan with the patient to explain drug intake (IMiDs and corticosteroids): number and dose of tablets or capsules, daily self-administration times, and times in the month for stopping intake in discontinuous regimens. A monthly calendar included the discontinuous regimen of each of the drugs. The others potential additional drugs were included in the treatment plan. Then the nurse and the pharmacist trained the patient to manage serious ADR with simulation-based learning. The program focused on 3 clinical situations about IMiDs toxicity according to their potential severity and to the capacity of the patient to easily recognize them: febrile neutropenia, deep-vein thrombosis and pulmonary embolism. An interactive serious game was created as an educational tool comprising 3 scenarios. The first scenario described the symptoms of febrile neutropenia and VTE; the second was dedicated to prevention; and the last focused on the warning symptoms that should lead the patient to contact the physician promptly because of potential severity (fever, breathing difficulties, swollen and painful leg). The scenes were set, and different actions were proposed to the patient. Green or red lights indicated a correct or false answer. At the end of the session, a booklet was given to the patient, summarizing the treatment plan and the main information on ADR.

### Program evaluation

This prospective before-and-after study evaluated implementation of the SCARMET educational intervention, focusing on IMiDs. Additional drugs were not included in the evaluation. Due to the lack of validated tools, a specific questionnaire was developed. The first part assessed knowledge of IMiD hematologic and thromboembolic ADR, with 8 questions targeting blood cells, complete blood counts, anti-thrombotic treatment, clinical signs associated with neutropenia, thrombocytopenia and thrombosis. This assessment of knowledge was combined with patient self-assessment of certainty on a 6-point scale from 0 to 100%. The second part of the questionnaire investigated knowledge of self-management skills in case of febrile neutropenia (fever), deep-vein thrombosis (painful and swollen leg) and pulmonary embolism (shortness of breath and difficulty breathing). The skills assessment used two 4-item Likert scales: firstly, for the patient, 1- “It's hard for me to know what to do”, 2- “I do not feel comfortable handling this situation”, 3- “I think I know how to manage the situation and I can improve”, 4- “I know what to do”; and secondly for the nurse and pharmacist, 1- “The patient doesn’t identify the ADR and doesn’t know how to manage it”, 2- “The patient identifies the ADR but doesn’t manage it properly”, 3- “The patient doesn’t identify the ADR but manages it correctly”, and 4- “The patient identifies the ADR and manages it correctly". In both cases, the first two items correspond to unsatisfactory management of the ADR and the last two to satisfactory management. The patient's self-assessment was compared to the educational team’s assessment. The questionnaire was pre-tested with 10 patients to ensure correct understanding.

### Outcomes

Three face-to-face standardized interviews between patient and educational team were conducted to assess patients’ knowledge of IMiDs ADR and management skills. The first interview aimed to evaluate the knowledge of the patient. It occurred at the inclusion of the patient after a consultation with the hematologist (T0). The educational intervention was planned concurrently to the next appointment around one month after. The other two interviews aimed to evaluate the impact of the program. They were respectively planned one month (T1) and six months (T6) after the educational intervention, concurrently with hematologist consultation. Patients did not have to come back to the hospital for any of the SCARMET interventions.

Secondary objectives were to assess impact of SCARMET intervention on adherence and treatment satisfaction. Adherence was evaluated with the medication possession ratio (MPR). In France, IMiDs were dispensed by hospital pharmacists, so the MPR was calculated from hospital dispensing data, as the total days’ supply of IMiDs during the follow-up period divided by the number of days of the same period. The follow-up period was 365 days (6 months before and 6 months after the educational intervention) [19]. Only patients treated with IMiDs 6 months before and after the educational session were included in the MPR calculation. Patients with 80% or more MPR were considered as adherent as usually recommended [20]. Patients’ satisfaction with treatment was evaluated with the self-administered questionnaire SATMED-Q® at T0, T1 and T6. This questionnaire of seventeen questions asks about six criteria: efficacy, side effects, comfort of use, general opinion of the patient, effect of the treatment on the daily life and quality of monitoring provided by health professionals. Responses are expressed using a 5-point likert scale [21, 22].

### Statistical analysis

Descriptive statistics were collected for quantitative and categorical variables. The patient’s knowledge (correct answers and degree of certainty) and skills (competence in managing ADR) were compared between baseline and 1 and 6 months after the educational intervention. Categoric variables were expressed as percentages and compared on chi^2^ test or McNemar test as appropriate. Normally distributed data were analyzed on paired t-test. Analyses used MedCalc® software (MedCalc Software, Ostend, Belgium). Differences were considered significant at a *p-*value ≤ 0.05.

## Results

### Population characteristics

**Fig 1:**
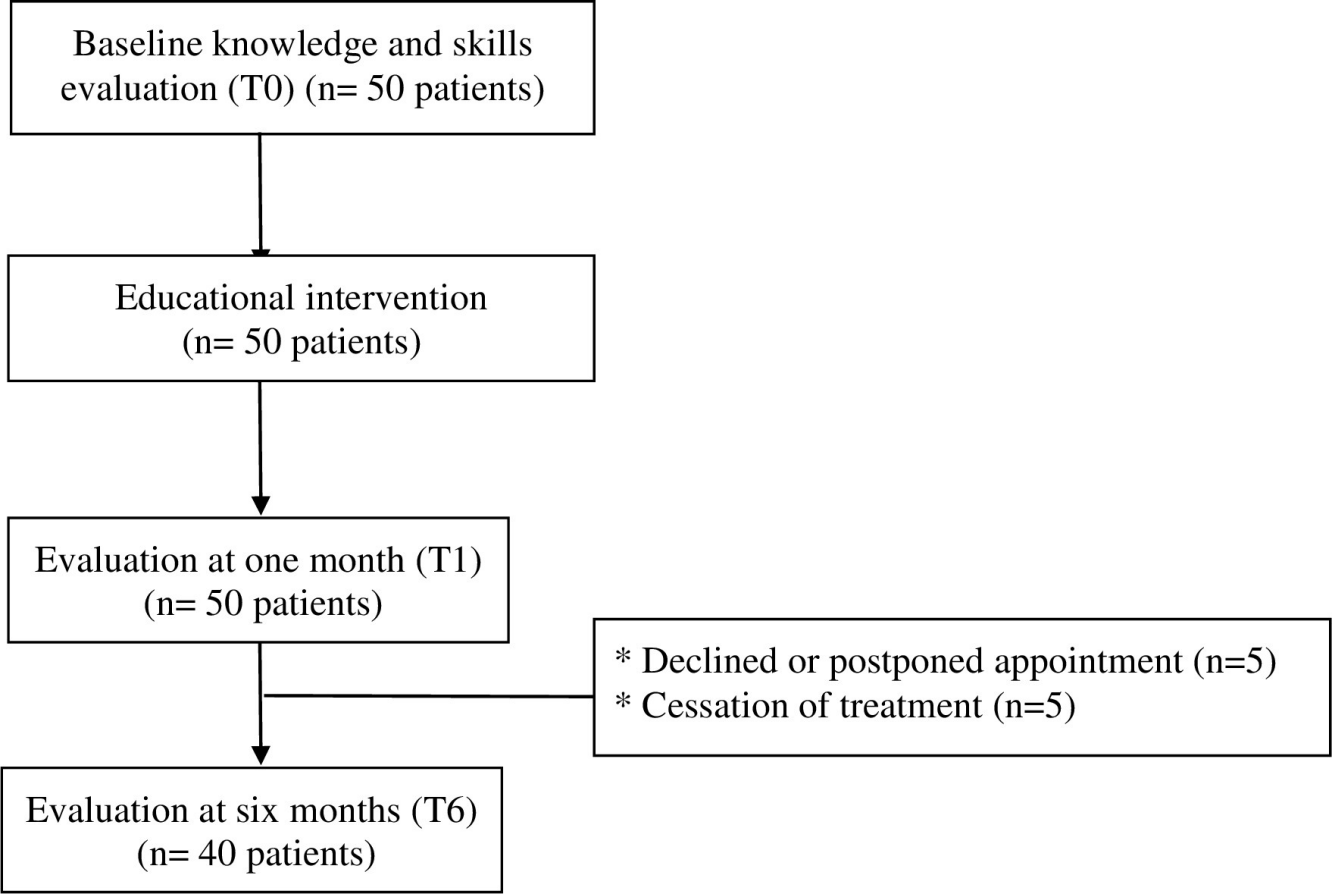
Study flow chart. T0: baseline, T1: 1 month after educational session,T6: 6 months after educational session.

Fifty consecutive multiple myeloma patients with IMiDs were enrolled (Table 1). All patients received the educational session. Forty of them underwent the 6-month evaluation (Fig 1); the other 10 either declined or postponed the appointment (n = 5), or had treatment cessation for remission (n = 1), disease progression (n = 3) or hematologic toxicity (n = 1).

**Table 1:** Patient characteristics.

	All patients (n = 50)
**Patients**	
Gender, n (%)	
Male	34 (68)
Female	16 (32)
Age (years)	
Mean ± SD	67 ± 7,2
Median (range)	67 (50–84)
Marital status, n (%)	
Living together: Married/ partnership	40 (80)
Living alone: Divorced/single/widowed	10 (20)
Occupational status, n (%)	
Working	6 (12)
Not working (without employment, retired, unemployed)	44 (88)
Level of education, n (%)	
Lower educational status (≤ high school certificate)	33/47 (70)
Higher educational status (> high school certificate)	14/47 (30)
**Multiple myeloma**	
Time from diagnosis to inclusion (months)	
Mean ± SD	73 ± 50
Median	70
Range	1–183
**Treatment regimen**	
IMiDs, n (%)	
Thalidomide	8 (16)
Lenalidomide	37 (74)
Pomalidomide	5 (10)
Line of therapy, n (%)	
First-line	8 (16)
Second-line	17 (34)
≥ third-line	25 (50)
Duration of IMiD treatment (months)	
Mean ± SD	14 ± 20
Median (range)	7 (1–82)
Previous IMiD treatment, n (%)	17 (34)

The mean age was 67 years +/- 7.2 (range, 50–84 years); 34 patients (68%) were male (Table 1). Mean duration of IMiD treatment before enrollment was 14 months +/- 20 (range, 1–82 months).

### Patients’ knowledge of ADR

The overall level of knowledge at baseline was poor in the study population (n = 50). One quarter of the population knew the term leukocytes (28%) and 30% knew the role of blood counts. Three-quarters reported that they did not know the role of neutrophils (74%). Less than half (42%) mentioned fever or infection in case of neutropenia, and only 36% quoted epistaxis or unexplained hematoma in case of thrombopenia. Sixty-for percent cited shortness of breath or swollen painful leg in the event of blood clot formation.

One month after the intervention (n = 50 patients), the number of correct answers and the rate of ≥80% confidence in knowledge both increased (p<0.001) over baseline. The 6-month results (n = 40 patients) were no longer significantly better than baseline, except for knowledge of clinical manifestations of neutropenia, which remained higher. The rate of correct answers and correct answers with ≥80% certainty remained higher than baseline (p<0.001), but there is a loss of acquired knowledge since the 1-month evaluation (p<0.05) (Tables 2 and 3).

**Table 2:** Knowledge at baseline (T0, n = 50 patients), 1 month (T1, n = 50 patients) and 6 months (T6, n = 40 patients).

	Patients with 1 month evaluation (n = 50)			Patients with 1 and 6 month evaluation (n = 40)			
Topic Number and percentage of patients with correct answers Mean degree of certainty (DC)	T0	T1	p-value T0/T1	T0	T1	**T6**	**p value T0/T6**
**blood cells**							
**n, %**	36 (72%)	45 (90%)	**= 0.01**	29 (72%)	*39 (97%)*	37 (92%)	>0.05
**Mean DC**	56	78	**<0.01**	56	*88*	75	**<0.05**
**Significance of « leukocytes »**							
**n, %**	14 (28%)	31 (62%)	**<0.001**	13 (32%)	*25 (62%)*	18 (45%)	>0.05
**Mean DC**	24	57	**<0.001**	28	*86*	42	**= 0.01**
**role of neutrophils**							
**n, %**	13 (26%)	21 (42%)	>0.05	9 (22%)	*17 (42%)*	13 (32%)	>0.05
**Mean DC**	22	38	**<0.05**	19	*87*	32	**<0.05**
**role of blood count**							
**n, %**	15 (30%)	25 (50%)	**<0.05**	12 (30%)	*21 (52%)*	14 (35%)	>0.05
**Mean DC**	28	48	**<0.05**	28	*91*	32	>0.05
**anti-thrombotic treatment**							
**n, %**	42 (84%)	48 (96%)	>0.05	38 (95%)	*39 (98%)*	33 (83%)	>0.05
**Mean DC**	83	90	>0.05	83	92	87	>0.05
**clinical signs of neutropenia**							
**n, %**	21 (42%)	42 (84%)	**<0.001**	16 (40%)	*34 (85%)*	28 (70%)	**<0.05**
**Mean DC**	41	74	**<0.001**	40	*89*	56	**<0.05**
**clinical signs of thrombopenia**							
**n, %**	18 (36%)	31 (62%)	**<0.01**	12 (30%)	*23 (57%)*	19 (47%)	>0.05
**Mean DC**	32	52	**<0.001**	30	*75*	39	>0.05
**clinical signs of formation of blood clots**							
**n, %**	32 (64%)	42 (84%)	**<0.05**	26 (65%)	*34 (85%)*	33 (82%)	>0.05
**Mean DC**	42	80	**<0.001**	39	*93*	59	**<0.01**

T0: baseline, T1: 1 month after educational session,T6: 6 months after educational session, DC: degree of certainty evaluated with a 6-point scale from 0 to 100%.

**Table 3:** Answers at baseline (T0, n = 400), 1 month (T1, n = 400) and 6 months (T6, n = 320).

Percentage of answers	T0 (n = 400)	T1 (n = 400)	p value T0/T1	T0 (n = 320)	*T1 (n = 320)*	T6 (n = 320)	p value T0/T6
**Correct answers**	48%	71%	**<0.001**	48%	*72%*	61%	**<0.001**
**Incorrect answers**	8%	2%	**<0.001**	8%	*2%*	7%	>0.05
**Unknown answers**	44%	27%	**<0.001**	44%	*26%*	32%	**<0.005**
**Correct answers with DC ≥ 80%**	33%	61%	**<0.001**	32%	*63%*	48%	**<0.001**
**Correct answers with DC < 60%**	15%	9,5%	**<0.001**	16%	*9%*	13%	>0.05
**Incorrect answers with DC < 60%**	7.0%	1.2%	**<0.001**	6.5%	*1*.*5%*	5.3%	>0.05
**Incorrect answers with DC ≥ 80%**	1.2%	0.7%	>0.05	1.5%	*0*.*9%*	1.9%	>0.05

T0: baseline, T1: 1 month after educational session,T6: 6 months after educational session.

DC = degree of certainty evaluated with a 6-point scale from 0 to 100% / n = 400 answers at T0 and T1 (i.e. 8 knowledge questions for 50 patients) / n = 320 answers at T6 (i.e. 8 knowledge questions for 40 patients).

T0/T1 comparison: concern the 50 patients and T0/T6 comparison: concern the 40 patients who completed the study.

### Patients’ knowledge of self-management skills

The patients’ ability to manage serious ADR at baseline (n = 50) was moderate. Half (48%) would neither have taken their temperature nor contacted their physician in case of fever. Thirty percent would not alert the physician or call the emergency number in the event of warning symptoms of VTE. These patients were considered by the clinical pharmacist as non-competent to manage these potential ADR. The percentage of patients unable to link a clinical manifestation to a potentially serious ADR was 56% for fever and neutropenia, 26% for lower-limb swelling and deep-vein thrombosis, and 66% for abrupt breathlessness and pulmonary embolism. Moreover, correct identification of an ADR was not systematically followed by appropriate management. Although some patients correctly identified the warning symptoms, others would not have managed the occurrence of complications as febrile neutropenia (10 patients), deep vein thrombosis (6 patients) or pulmonary embolism (3 patients). They would not have taken their temperature or called their doctor or the emergency number if these situations had occurred.

At the 1-month evaluation (n = 50), the number of patients with self-care skills deemed satisfactory by the pharmacist increased: from 26 to 43 (p<0.001) for febrile neutropenia, 35 to 48 (p<0.01) for pulmonary embolism, and 35 to 49 (p<0.01) for phlebitis (Table 4). Pharmacist evaluation at 6 months (n = 40) reported that the number of patients with satisfactory self-care skill level was greater than at baseline (Table 4) and almost as high as on the 1-month evaluation. The number of patients competent to manage febrile neutropenia, pulmonary embolism and deep-vein thrombosis respectively increased from 18 to 36 (p<0.001), 29 to 39 (p<0.01) and 26 to 38 (p<0.01). In all, 90% to 98% of patients reacted correctly in case of fever, of swollen and painful lower limb and of breathlessness (Table 4).

**Table 4:** Self-management skills at baseline, 1 month (n = 50) and 6 months (n = 40).

Patients evaluated by the pharmacist as competent for managing ADRs	T0 (n = 50)	T1 (n = 50)	p value T0/T1	T0 (n = 40)	*T1 (n = 40)*	T6 (n = 40)	p value T0/T6
**Febrile neutropenia (n,%)**	26 (52%)	43 (86%)	**<0.001**	18 (45%)	*34 (85%)*	36 (90%)	**<0.001**
**Pulmonary embolism (n,%)**	35 (70%)	48 (96%)	**<0.01**	29(73%)	*39 (98%)*	39 (98%)	**<0.01**
**Deep-vein thrombosis (n,%)**	35 (70%)	49 (98%)	**<0.01**	26 (65%)	*40 (100%)*	38 (95%)	**<0.01**

ADR: adverse drug reaction.

T0: baseline, T1: 1 month after educational session,T6: 6 months after educational session.

T0/T1 comparison: concern the 50 patients and T0/T6 comparison: concern the 40 patients who completed the study.

### Adherence

Forty patients were treated with IMiDs 6 months before and after the educational session. Thirty-seven patients (97%) were adherent with MPR ≥ 80% within 12 months follow-up period: MPR value was 100% for 32 patients, 92 ± 1.7% for 5 patients and 67% for 1 patient. The latter had a MPR < 80% before and after the educational session. There was no significant difference between the median MPR over the period of 6 months before and after the educational intervention.

### Treatment satisfaction with medicines

Mean satisfaction scores did not differ significantly from baseline (60/100) at 1 and 6-month evaluation (61/100; p> 0.05). The items for which the patients were least satisfied in the self-administered questionnaire concerned ADR, with gastro-intestinal disorders, neuropathy and fatigue which appeared to have a major impact on physical, leisure and/or daily living activities. Patients were satisfied with comfort of use, follow-up and the information provided by the healthcare professionals.

## Discussion

The SCARMET program, an educational intervention of the real life multidisciplinary plan for cancer outpatients ONCORAL, seems to be effective in improving knowledge of self-care skills in multiple myeloma outpatients treated with IMiDs. The educational objectives focused on self-identification and management of fever and thromboembolic clinical symptoms suggestive of febrile neutropenia and VTE.

The increased skills level reached at 1-month post-intervention appeared to be maintained at 6 months. However, there was partial loss of knowledge at 6 months, suggesting that the program needs to be optimized, with more closely spaced interventions. In this study, the educational intervention was performed at any time during IMiD treatment. The interval between initiation of IMiDs and the educational session can have an influence on the patient's ability to memorize the information, receptivity and on the acceptability of the educational session. Molassiotis et al. demonstrated that an educational session in self-management of ADR during the first two cycles of treatment had the greatest impact in reducing toxicity [23]. On the other hand, providing information at initiation of treatment can lead to a burden of information that is difficult to assimilate for patients. Further information in the first few weeks seems also to be an appropriate and useful approach, with improved continuity of care and a more positive treatment experience for patients. These results are consistent with those of other studies showing that, regardless of age, cancer patients forget substantial amounts of information. Jansen et al. showed that almost 50% of disease and treatment information was remembered by cancer patients 10 days after consultation with the oncologist [24]. In this study, the amount of information given to the patient is limited and selected by the panel of experts in view of the ADR potential seriousness and the possibility for the patient to act on the situation. Febrile neutropenia remains infrequent in patients with IMiDs in real life [25]. However its readily observable signs and its serious consequences lead the panel of experts to regard it as a warning symptoms in multiple myeloma patients. The use of the MASCC Oral Agent Teaching Tool (MOATT©) [26] could have been interesting to complete the provided informations about treatment (storage, handling and disposal…) and others ADR (gastrointestinal disorders, peripheral neuropathy…). This tool should be considered for further educational intervention. The written support given to the patient at the end of the SCARMET educational session was possibly useful for improving recall of treatment information. Other suggestions are, for example, to tailor information to individual needs [27] and to repeat the information [28]. In order to limit the number of consultations in the hospital, phone calls, connected device or a website can also contribute to follow-up [29, 30].

Patients’ confidence in their knowledge was evaluated as degrees of certainty on the double Likert scale for the educational team and patient. The SCARMET intervention seemed to improve patients’ self-evaluated confidence. Previous studies reported self-assessment to be a formative evaluation process that allows the patient to take a critical look at his or her own decision-making concerning ADR management [14, 15]. The double evaluation, by patient and health-care professional, facilitates an exchange of point of views, especially in case of discordant judgments. These outcomes should be taken into account in planning further educational sessions: knowledge with low certainty could be managed by repeated educational interventions; persistent wrong convictions after the education session would require a stronger educational effort, especially if a high degree of certainty is associated, since it is well known that educational sessions need to be repeated to maintain a high level of knowledge [31].

This study used a before / after design to show the impact of therapeutic education on the patient's level of knowledge, as an intermediate endpoint and based on the assumption that knowledge conditions patient behavior. These results are important as it has been reported that the patients’ knowledge of the drugs is associated positively with adherence [32] and would increase their active participation in treatment [17]. However the change in knowledge alone does not guarantee the change in health behavior. Further studies need to evaluate the reduction of clinical events associated with the identification (time to recognition of VTE…) and the management of ADR (time from first temperature to antibiotics…) by the patient to confirm this hypothesis. Recent studies demonstrated that early patient-reported symptom monitoring may be useful as part of high-quality cancer care, with significantly less emergency consultation and admission and positive impact on survival [33].

The management of IMiD ADR by cancer outpatients themselves is essential in order to minimize impact on quality of life and on adherence to treatment. Interruption of treatment with the aim of reducing ADR is also reported to be a major cause of intentional non-adherence [34]. Numerous educational approaches have been developed in cancer care to optimize adherence and ADR management [35, 36], but have been little implemented in multiple myeloma. Nevertheless, IMiDs are associated with at least 3 risk factors for non-adherence: long-term treatment, complex treatment plan with dexamethasone (discontinuous cycles), and potentially serious ADRs [37]. Using the MPR, most patients were found to be adherent to IMiD treatment. Different tools have been developed and validated in order to effectively and accurately assess adherence in a wide range of diseases, but there is still no “gold standard” method. Self-completed questionnaires, although validated, offer subjective answers with a risk of overestimation [38]. MPR does not automatically constitute proof of drugs intake, which may also give overestimated adherence. However MPR constitutes an objective criteria based on accurate pharmacy records and offers information on the possession of medication [39]. The rate of adherence in the present study (97% patients with MPR ≥ 80%) was similar to recent studies [11, 12, 40]. Against all expectations and contrary to other populations of cancer patients [41, 42], IMiDs treatment adherence does not appear a significant issue.

The patient's satisfaction with treatment was explored because of the positive correlation between satisfaction and knowledge regarding treatment [43]. Such patient reported outcomes is important since several studies reported a significant correlation between patient’s satisfaction and adherence in patients with chronic pathologies [21] and more recently in cancer patients with oral chemotherapy [44]. However in the present study, patient satisfaction was moderate and remained constant throughout the study period.

Relevance for clinical practice of these results have to be discussed. The early recognition and the appropriate management of ADR represent a key challenge for clinicians caring for outpatients with multiple myeloma. This study pointed that a significant number of patients did not know what to do when suspecting a blood clot or febrile neutropenia and highlighted the need for intervention. This multidisciplinary program was easily integrated into the patients care program and appears to be effective to improve patient’s knowledge. This approach is important as we known that teaching patients the warning signs, especially VTE, and their management is potentially life-saving [45].

The study limitations concerned the single-center non-controlled before-after design so we can only suppose that the gained knowledge is a consequence of the SCARMET educational program. The psychometric properties of the SCARMET questionnaire to assess knowledge of hematological ADR and self-management (sensitivity, validity, fidelity) need to be determined. The use of subjective degree of certainty should likewise be validated in multiple myeloma patients, as it depends on several factors such as individual self-estimation capacity and self-confidence. Such biases were partially controlled, as the questionnaire was administered to the same patients before and after the educational intervention. It is also necessary to ensure that targeted education on hematological and VTE does not reduce the patient's involvement in the detection of other ADR.

## Conclusion

The SCARMET program is the first to be developed by the ONCORAL platform dedicated to care and research to enhance the safety of oral chemotherapy in cancer outpatients. The present study highlighted the need to inform myeloma outpatients about the management of potentially IMiDs toxicities. The results suggested that a single educational session improved knowledge of IMiDs adverse effects self-management skills. The partial loss of knowledge over time was a predictable result, demonstrating the interest of repeated educational sessions. These results strongly encourage the development of other multidisciplinary follow-up of outpatients treated by oral anticancer drugs in real life.

## Supporting information

S1 File (DOCX)

S2 File (DOCX)
