# Supplementary material for: Immunomodulatory drugs in multiple myeloma: Impact of the SCARMET (Self CARe and MEdication Toxicity) educational intervention on outpatients’ knowledge to manage adverse effects
Source: PLoS One. 2020 Dec 4;15(12):e0243309. doi: 10.1371/journal.pone.0243309 (PMC7717911; doi:10.1371/journal.pone.0243309)
Supplement: S2 File — (DOCX) [file pone.0243309.s002.docx]

*Dear Madam, Dear Sir,*

*Thank you for agreeing to participate in this study.*

*First of all, I will ask you a few questions about your treatment with ........................*

*Then we will reflect on what to do in case of adverse events.*

*For some questions, I will ask you to indicate on this slide if you are rather sure of your answer or not.*

*The estimated time to answer this questionnaire is 15 minutes.*

1. **What has your doctor told you about your treatment?**

**2. What did the doctor tell you about the side effects of this treatment?**

*Patient did not mention effects on blood*

*Patient mentioned effects on blood*

*You did not mention the effects on the blood, yet this treatment can affect healthy blood cells.*

*You started talking about the effects on the blood. Indeed, it can sometimes happen that this treatment acts on healthy blood cells*

**3. Can you name the three types of blood cells?**

**4. What does the term "leukocytes" mean?**

**5. Have you heard about neutrophils?** **If so, what is their role?**

**6. What is the test that monitors blood cells?**

**7. If the treatment lowers your white blood cells, how can this happen?**

**8. If the treatment reduces your platelets, how can this happen?**

**9. Can the treatment cause blood clots?**

**10. If a blood clot forms, how can it happen?**

**11. Is medication prescribed to reduce the risk of blood clots? If so, which one?**

**12. What situations would lead you to call your doctor quickly?**

**Role-playing**

**1. You are at home and are experiencing unusual shortness of breath with pain in your chest without having made a special effort.** **You then think of a side effect of your treatment, which one?** **What is your reaction?**

**2. One morning when you wake up you think you have a fever or signs of infection. You then think of a side effect of your treatment, what could it be due to? What is your reaction?**

**3. For no particular reason, you feel intense pain in your calf.** **You then think of a side effect of your treatment, which one?**  **What is your reaction ?**
