# Supplementary material for: Immunomodulatory drugs in multiple myeloma: Impact of the SCARMET (Self CARe and MEdication Toxicity) educational intervention on outpatients’ knowledge to manage adverse effects
Source: PLoS One. 2020 Dec 4;15(12):e0243309. doi: 10.1371/journal.pone.0243309 (PMC7717911; doi:10.1371/journal.pone.0243309)
Supplement: Supplementary file 2 [file pone.0243309.s001.docx]

My immuno-modulator treatment

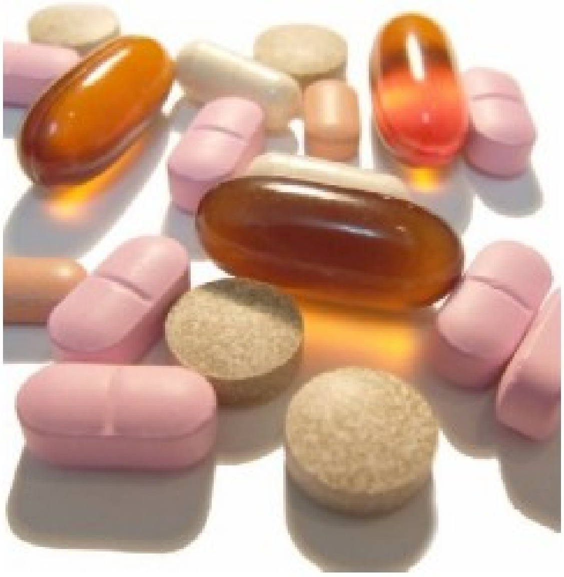

The side effects of my treatment by ....

➢ My treatment can lower my white blood cells.

✓ *What can I feel?*

Fever, chills, sweating, respiratory symptoms: coughing, spitting…

✓ *What should I do if these signs appear?*

I take my temperature

I call the doctor if my temperature > 38°C and/or if I experience these symptoms

➢ My treatment can lower my platelets

call the doctor in case of heavy bleeding and/or unexplained heavy bruising.

➢ My treatment may cause blood clots to form.

✓ *What can I feel?*

Swollen leg, painful, red (Phlebitis / thrombosis?)

Chest pain, difficulty breathing, sudden shortness of breath (Pulmonary embolism?)

Paralysis on one side of the body (arm or leg), visual or speech disorders (Cerebrovascular accident?).

I call the doctor and/or 15 or 112 if I experience these symptoms

If any other unusual symptoms occur, I contact the doctor.

Hospices Civils de Lyon

Medications	Morning	Noon	Evening	Bedtime	Duration of treatment	
					1 2 3 4<br>5 6 7 8 9 10 11<br>12 13 14 15 16 17 18<br>19 20 21 22 23 24 25<br>26 27 28 29 30 31	1 2 3 4<br>5 6 7 8 9 10 11<br>12 13 14 15 16 17 18<br>19 20 21 22 23 24 25<br>26 27 28 29 30 31
					1 2 3 4<br>5 6 7 8 9 10 11<br>12 13 14 15 16 17 18<br>19 20 21 22 23 24 25<br>26 27 28 29 30 31	1 2 3 4<br>5 6 7 8 9 10 11<br>12 13 14 15 16 17 18<br>19 20 21 22 23 24 25<br>26 27 28 29 30 31
					1 2 3 4<br>5 6 7 8 9 10 11<br>12 13 14 15 16 17 18<br>19 20 21 22 23 24 25<br>26 27 28 29 30 31	1 2 3 4<br>5 6 7 8 9 10 11<br>12 13 14 15 16 17 18<br>19 20 21 22 23 24 25<br>26 27 28 29 30 31
					1 2 3 4<br>5 6 7 8 9 10 11<br>12 13 14 15 16 17 18<br>19 20 21 22 23 24 25<br>26 27 28 29 30 31	1 2 3 4<br>5 6 7 8 9 10 11<br>12 13 14 15 16 17 18<br>19 20 21 22 23 24 25<br>26 27 28 29 30 31
					1 2 3 4<br>5 6 7 8 9 10 11<br>12 13 14 15 16 17 18<br>19 20 21 22 23 24 25<br>26 27 28 29 30 31	1 2 3 4<br>5 6 7 8 9 10 11<br>12 13 14 15 16 17 18<br>19 20 21 22 23 24 25<br>26 27 28 29 30 31
					1 2 3 4<br>5 6 7 8 9 10 11<br>12 13 14 15 16 17 18<br>19 20 21 22 23 24 25<br>26 27 28 29 30 31	1 2 3 4<br>5 6 7 8 9 10 11<br>12 13 14 15 16 17 18<br>19 20 21 22 23 24 25<br>26 27 28 29 30 31
					1 2 3 4<br>5 6 7 8 9 10 11<br>12 13 14 15 16 17 18<br>19 20 21 22 23 24 25<br>26 27 28 29 30 31	1 2 3 4<br>5 6 7 8 9 10 11<br>12 13 14 15 16 17 18<br>19 20 21 22 23 24 25<br>26 27 28 29 30 31

Hospices Civils de Lyon

Tips

- ✓ Maintain good mobility, exercise regularly (walking, etc.).
- ✓ Drink enough water during the day
- ✓ Ask my doctor or pharmacist for advice before taking any treatment.

Coordonnées utiles

Haematologist:

……………………………………………
……………………………………………
……………………………………………
……………………………………………

General Practitioner

……………………………………………
……………………………………………
……………………………………………
……………………………………………

Pharmacy

……………………………………………
……………………………………………
……………………………………………
……………………………………………
